# Acute hemorrhagic pancreatitis following influenza infection: a case report 

**DOI:** 10.1186/s13256-023-03906-0

**Published:** 2023-05-03

**Authors:** Chathula Ushari Wickramasinghe, Murugapillai Sivasubramanium, Rohitha Muthugala

**Affiliations:** 1 JMO’s Office, National Hospital Kandy, Kandy, Sri Lanka; 2 Virology Laboratory, National Hospital Kandy, Kandy, Sri Lanka

**Keywords:** Maternal death, Acute hemorrhagic pancreatitis, Influenza A virus, Case report

## Abstract

**Background:**

Acute hemorrhagic pancreatitis is a life-threatening condition leading to shock and multiorgan failure. Although prevalent in the general population, the incidence during pregnancy is low, with a high maternal and fetal mortality rate. The highest incidence is in the third trimester/early postpartum period. Infectious etiology for acute hemorrhagic pancreatitis is rare with only a handful of cases following influenza infection being documented in the literature.

**Case presentation:**

A 29-year-old Sinhalese pregnant lady in the third trimester presented with an upper respiratory tract infection and abdominal pain, for which she was managed with oral antibiotics. An elective caesarean section was done at 37 weeks gestation due to a past section. On postoperative day 3 she developed a fever with difficulty in breathing. Despite treatment, she succumbed to death on the sixth postoperative day. The autopsy revealed extensive fat necrosis with saponification. The pancreas was necrosed and hemorrhagic. The lungs showed features of adult respiratory distress syndrome and necrosis was observed in the liver and kidneys. Polymerase chain reaction of lungs detected influenza A virus (subtype H3).

**Conclusion:**

Although rare, acute hemorrhagic pancreatitis from an infectious etiology carries risk of morbidity and mortality. Therefore, a high level of clinical suspicion must be upheld among clinicians to minimize adverse outcomes.

## Introduction

According to the Sri Lankan national maternal mortality review 2016, necrotizing pancreatitis has been identified as a rare unpreventable cause of maternal death [1]. Considering global statistics, pancreatitis is documented to have an estimated prevalence of 0.00008–0.1% in pregnancy. Although extremely rare, it has been identified as a major cause of high morbidity and mortality during pregnancy, with a myriad of adverse effects on the mother and the fetus [2, 3]. According to the literature, pancreatitis in pregnancy is more commonly observed in multiparous females in the third trimester and the early postpartum period, with the incidence increasing with advancing gestation. The commonest causes have been identified as gallstone disease, hyperlipidemia, and alcohol abuse [2]. Rarely, infective agents, including the influenza virus, have been observed to cause acute pancreatitis [4–8].

## Case report

**Fig. 1:**
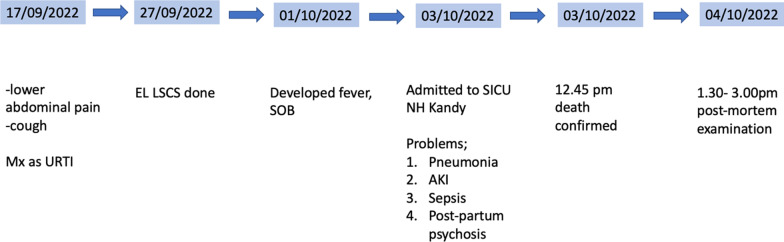
Clinical timeline. *URTI* upper respiratory tract infection, *EL LSCS* elective lower segment cesarean section, *SOB* shortness of breath, *SICU* surgical intensive care unit, *NH Kandy* National Hospital Kandy, *AKI* acute kidney injury

The study subject in our case is a 29-year-old Sinhalese pregnant female whose past medical and surgical history was unremarkable. She was in her third pregnancy with one living child and the first pregnancy had ended in a first-trimester miscarriage. At 36 weeks of gestation, she presented to the local hospital with a dry cough and abdominal and back pain. The pregnancy up to that point had been unremarkable. She was managed for an upper respiratory tract infection with oral antibiotics and symptomatic treatment. She developed fever spikes with elevated inflammatory markers during the ward stay. She underwent an elective cesarean section under spinal anesthesia and delivered a live baby. The surgery and recovery were unremarkable. On the second postoperative day, she developed diarrhea (around ten times/day) with colicky abdominal pain. Symptomatic treatment with fluid resuscitation was done. Based on the clinical presentation and epidemiology pattern influenza viral infection was suspected and oseltamivir 75 mg 12 hourly was started. Her clinical condition gradually deteriorated with fluctuations in the level of consciousness and difficulty in breathing. Her blood investigations showed neutrophil leukocytosis and elevated inflammatory markers (C-reactive protein) with metabolic acidosis on blood gas analysis. Chest X-ray showed opacities in both lung fields indicative of bilateral lung consolidations. She was managed in the intensive care unit with intravenous antibiotics and supplementary oxygen. However, there was a progressive derangement in her organ functions with elevated serum creatinine and liver enzymes. A clinical diagnosis of sepsis following pneumonia was made. Abdominal ultrasound was unable to detect any sonographic evidence of intra-abdominal sepsis. Rapid antigen testing for the SARS-CoV-2 virus was performed but found to be negative. She was electively intubated and transferred to the National Hospital Kandy. Despite intensive resuscitation, she succumbed to death on postoperative day six (Fig. 1). An inquest was ordered, and a complete postmortem examination was conducted.

**Fig. 2:**
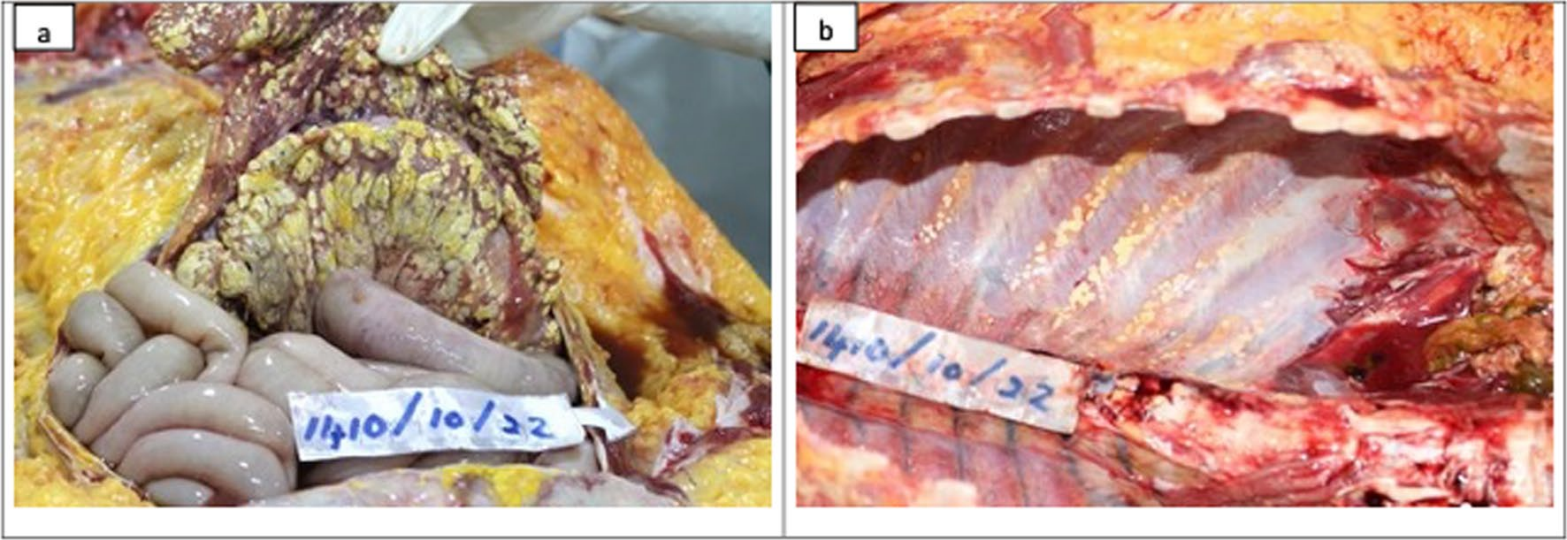
Postmortem findings: gross examination. During the internal examination, extensive fat necrosis and saponification was observed in the abdominal (**a**) and chest cavities (**b**)

**Fig. 3:**
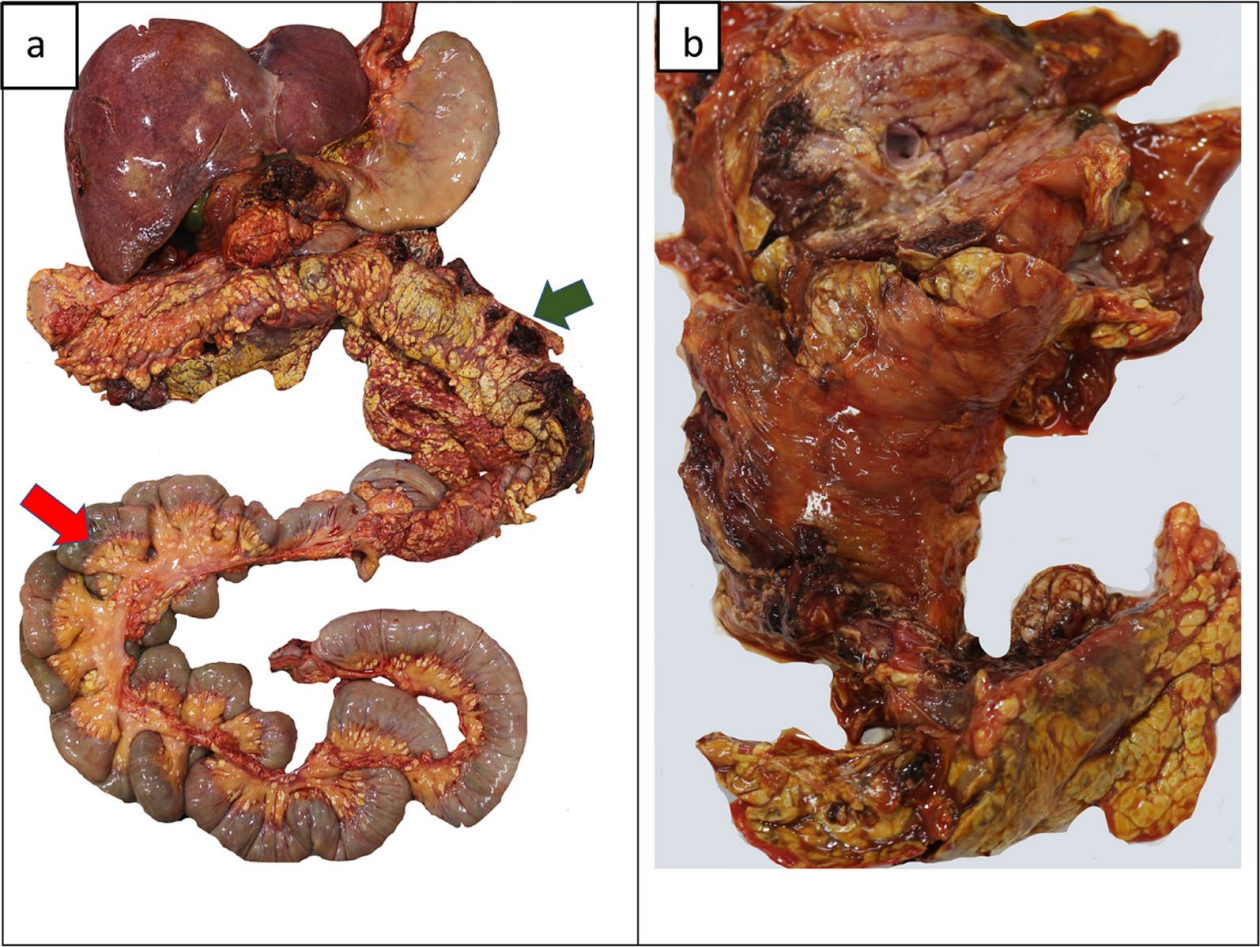
Postmortem findings: gross examination. **a** Hemorrhage into peripancreatic tissues (green arrow) with extensive saponification of omental adipose tissue (red arrow) and **b** hemorrhagic necrosis of the pancreas

An average-built young female with evidence of pregnancy and delivery was observed at the autopsy. Internal examination revealed extensive fat necrosis with saponification in the pleural and peritoneal cavities (Fig. 2). Around 500 ml of straw-colored fluid was in the abdominal cavity, and the peritoneum appeared unhealthy and dull. A mass of adhered bowel loops was noted at the left hypochondriac region adhered to the pancreas and peripancreatic tissue. Extensive hemorrhagic necrosis of the pancreas was noted (Fig. 3). Furthermore, features of adult respiratory distress syndrome (ARDS) of the lungs with bilateral pleural effusions, necrosis of the liver parenchyma, and edema of the kidneys with pale cortex and congested medulla were noted. The heart and brain were devoid of any gross pathology. Other possible causes for acute pancreatitis including gallstone disease were excluded during the post-mortem examination.

**Fig. 4:**
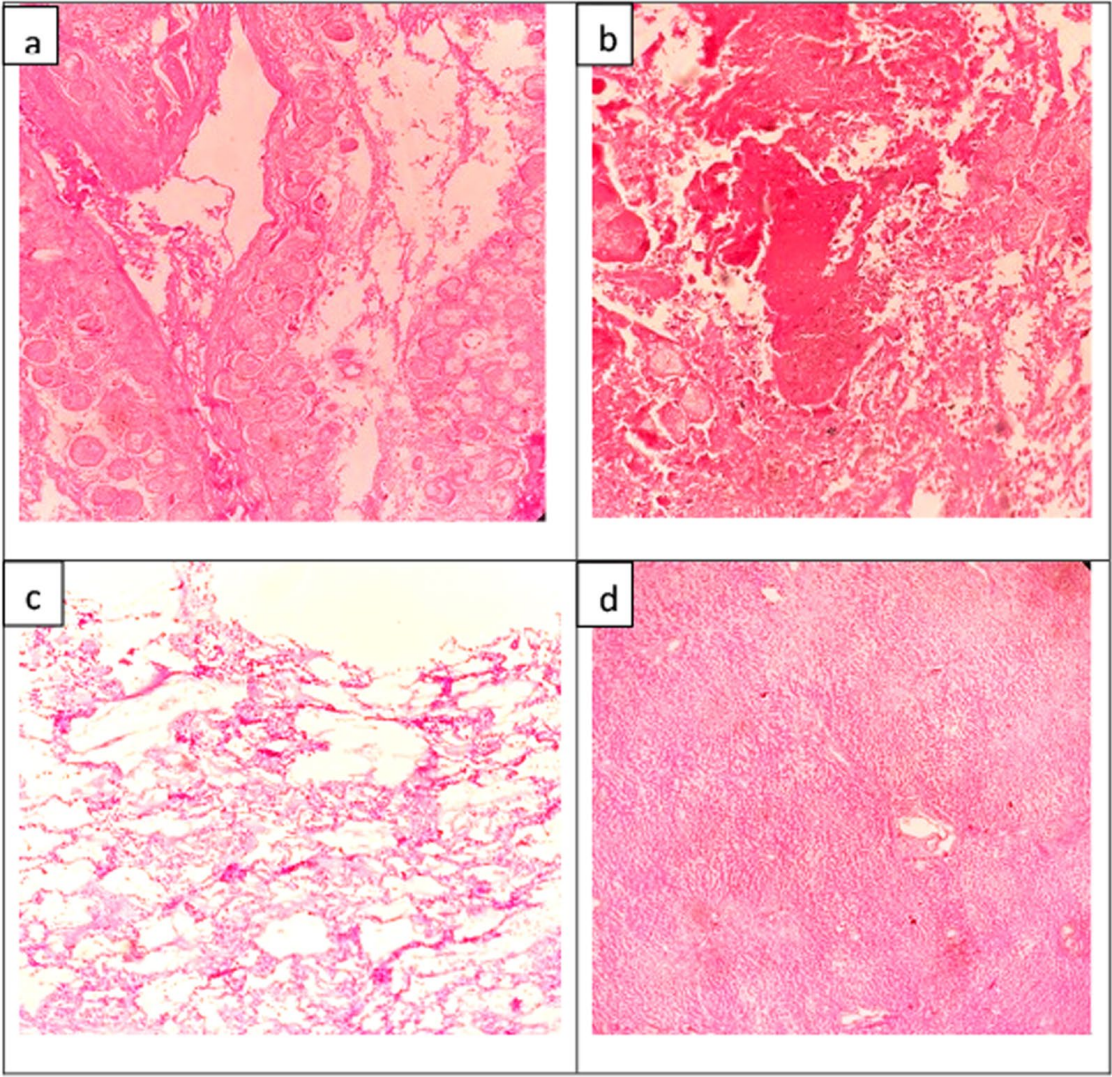
Histopathology (H&E stain). **a** and **b** necrosis of pancreatic cells and adipocytes with interstitial hemorrhages, **c** DAD of lungs with hyaline membrane formation and inflammatory infiltrate, **d** centri-venular necrosis in the liver

Influenza A virus ribonucleic acid (RNA) was detected in nasopharyngeal swabs and postmortem lung tissue by a commercial polymerase chain reaction (PCR) assay and was negative for other respiratory viruses (RespiFinder 2 Smart, PathoFinder, Netherlands). SARS-CoV-2 RNA was not detected in either sample by reverse transcription (RT)–PCR (RealStar, Altona Diagnostics, Germany). Further, the sample was referred to the National Influenza Centre and confirmed as subtype H3. Acute enterovirus, mumps, human immunodeficiency virus (HIV), cytomegalovirus, and herpes simplex virus infections were excluded by PCR and serology testing. Histology showed extensive hemorrhagic infarction of the pancreas with hemorrhagic fat necrosis in the surrounding adipose tissue (Fig. 4). The lungs showed diffuse alveolar damage with hyaline membranes and a polymorphonuclear inflammatory infiltrate in the alveolar spaces. Other organs showed hypoxic changes with acute tubular necrosis in the kidneys and centri-venular necrosis with mild fatty changes in the liver. The heart and brain were unremarkable histologically.

Written informed consent was obtained from the husband of the deceased for their anonymized information to be published in this article.

## Discussion

Infectious etiology has been documented in the literature as being responsible for around 10% of cases of acute pancreatitis, with a variety of causative agents including bacteria (*Mycoplasma*, *Legionella*, *Leptospira*, and *Salmonella*), fungi (*Aspergillus*), and parasites (*Toxoplasma*, *Cryptosporidium*, and *Ascaris*). Viruses such as mumps, HIV, coxsackie, hepatitis, and herpes simplex have been identified to be responsible for less than 1% of cases of acute pancreatitis [4]. Acute pancreatitis following influenza infection is extremely rare, with only a few reported cases from around the world [5–9].

In all these cases, early detection was achieved using radiological techniques such as ultrasonography and computed tomography. The management consisted of intravenous fluid resuscitation with a combination of antibiotics and antiviral treatment with oseltamivir. Although some cases were managed in the intensive care unit with noninvasive ventilation, no fatalities have been reported. Also, the literature has not reported fatal cases of influenza infection leading to acute pancreatitis during pregnancy.

Several *in vivo* and *in vitro* studies have been conducted to gain a better insight into the disease pathophysiology [10, 11]. In the mechanism of influenza A virus infection, sialic acid (SA) receptors, located on the surface of cells, act as an integral part by facilitating attachment and entry of viral particles into the cell. Using animal models, researchers have managed to demonstrate that SA receptors are expressed on the surface of pancreatic cells enabling the influenza A virus to attach and enter into the cells. Furthermore, it was observed that the influenza A virus is able to effectively infect and replicate inside pancreatic cells and induce cytopathic effects, leading to cell apoptosis and over-expression of cytokines and chemokines [10]. These findings explain the mechanism of pancreatic cell injury consequent to influenza A virus infection. Further studies have been able to establish this finding by demonstrating that certain influenza viruses have a tropism for pancreatic acinar cells [11]. These observations can be used to explain the manifestation of acute pancreatitis in influenza infection.

In the subject of our study, laboratory investigations confirmed a lower respiratory tract infection due to influenza A (subtype H3) virus. No other obvious evidence of a possible cause for acute pancreatitis was identified. In this case, despite early antiviral treatments, the rare complication of acute pancreatitis resulted in a fatal outcome.

### Limitations

Testing of pancreatic tissue for viral particles, which would have been useful in establishing the causal relationship, was not carried out in this case due to the limited resources in our hospital setting.

## Conclusion

Acute hemorrhagic pancreatitis during pregnancy is a rare occurrence but with a high risk of adverse maternal and fetal outcomes. The incidence during pregnancy has been observed to increase with advancing gestation. A variety of infective organisms including bacteria, viruses, fungi, and parasites have been identified as causative agents. Therefore, a high level of suspicion is needed among clinicians and pathologists regarding pregnant women presenting with abdominal pain.

## Data Availability

Not applicable.
